# Competitive Development Tools in Identifying Efficient Educational Interventions for Improving Pro-Environmental and Recycling Behavior

**DOI:** 10.3390/ijerph17010156

**Published:** 2019-12-24

**Authors:** Sorin Popescu, Diana Rusu, Mihai Dragomir, Daniela Popescu, Șerban Nedelcu

**Affiliations:** 1 Department of Design Engineering and Robotics, Technical University of Cluj-Napoca, 400114 Cluj-Napoca, Romania; sorin.popescu@muri.utcluj.ro (S.P.); mihai.dragomir@muri.utcluj.ro (M.D.); daniela.popescu@muri.utcluj.ro (D.P.); 2 Department of Finance, Accounting and Economic Theory, Transilvania University of Brasov, 500036 Brașov, Romania; serban_nedelcu@yahoo.de

**Keywords:** changing behavior, behavioral change theories, recycling behavior, sustainable wellbeing behavior, efficient educational interventions

## Abstract

Daily life in today’s dynamic world requires fast adaptation of people’s behavior to new challenges emerged from environmental, health, urban housing, transportation, etc. problems. A strong and complex tool in changing behaviors, education consumes significant resources and requires time for effective impact. The present study proposes a roadmap, integrating advanced methods for industrial competitive development (QFD) in configuring efficient educational interventions for changing people’s behavior on special thematic areas, such as environment and recycling. A literature review on applicable behavioral theories led to more than 30 behavioral factors (constructs) targetable by education, their specific impact evaluation being the subject of a survey among education professionals. Finally, to reach a mapping of efficient interventions with low costs, high impact and fast results criteria, educational interventions and behavioral factors were correlated by the QFD method on three dimensions addressing: teachers and trainers, contents and tools, respectively the national & institutional level. The current research results are focused particularly on changing recycling behaviors, but the overall concept and methodology can be expanded to incorporate any preoccupation in the areas of environment and health, as long-term drivers of public well-being.

## 1. Introduction

We are living in a strongly connected but also disrupted world, in a common house which all the members of the human family (regions, countries, groups and individuals) are meant to share. It is a house of both welfare and contrasts and inequalities, where defiant luxury coexists with deep poverty and brilliant knowledge discoveries live together with promiscuous and dangerous ignorance. It is the same house in which we all, poor and rich, educated or ignorant, are forced to share in tomorrow’s life and health the effects of our today’s behavior.

The population is growing fast [1] along with its expectations for prosperity and its daily consumption. Important changes in the social and age structure of the population [2], fast industrialization, increasing dependence on technology in all aspects of daily life, rapid and uncontrolled urbanization processes [3] together with continuous increase of industrial and household waste generation are just some of the emerging challenges of the current economic, social and environmental concerns, as well as of people’s daily lives. Efforts of world leading institutions are targeted towards defining and supporting roadmaps for this challenging future. The UN “2030 Agenda for sustainable development” [4] and other similar programmatic documents try to offer strategies, goals, policies and actions for a sustainable future. What is mentioned less or indirectly, but is essential in planning for a sustainable future is the people’s pro-future behavior (pro-environmental, pro-health, pro-humanity), the measure in which the people’s thinking and acting integrate this pro-global wellbeing behavior as a fundamental need and habit.

People‘s behavior is one of the most complex, diverse and difficult to master variables, especially when a change in a certain matter of large groups of people is needed or targeted. This issue provides the subject of many research studies, theories and models, structured specifically for their application field (health, sustainability, etc.) but using more or less common elements as basic constructs [5,6]. Such elements as knowledge, values, beliefs, attitudes, intentions etc. are indicated as precursors of people behaviors and as main targets for the interventions designed for their change.

Concerning possible interventions in this matter, they are mainly technical—targeting technologies and conditions favorable to the desired behavior, economical—using financial motivation and cultural – acting on the people’s way of thinking through education, information, etc. From the last category, providing information through all the channels including web has proved lately to be a strong tool (both with good and bad effects) in influencing (or manipulating) behaviors of large numbers of people [7,8,9] in environmental and health, commercial, political or other kind of decisions concerning issues generally limited to an area of application or in time. However, when behavior changes at large scale and long-term are targeted, educational interventions are largely accepted as unavoidable [10,11]. The aims of education are, generally speaking, far wider than to shape behaviors in a certain field, but when these behaviors are determinant for the wellbeing of large communities (regions, nations or even humanity), their modelling should be integrated in the core of broad educational interventions, designed on large scales and multiple levels and addressing long term needs to shape people’s interaction with reality. Relevant in this matter are those behaviors connected to sustainable development, that have become critical due to the increasing impact of human activities on the environment and climate and by their effects, on people’s wellbeing on long-term.

This need is visible in both rich and poor countries, communities or people, but the pressure is even higher on those lacking the knowledge and the material resources needed to undertake effectively this kind of demarche. In such situations, the adequacy of the concept of such an intervention should be evaluated not only in terms of its effectiveness—the extent to which it has the capacity to achieve its purpose, but also of its efficiency, the related costs becoming critical for its initiation and progress.

The present research proposes a structured approach for designing a thematic set of educational interventions, identifying its critical directions and actions from both the effectiveness’ and costs’ perspectives when dealing with education for sustainability, particularly targeting recycling issues. The meaning given by the present paper to educational interventions exceeds the one strictly related to teaching and learning elements such as contents, strategies or tools. It is considered here that for changing behaviors on long term, of large numbers of people and in important matters, these interventions have to be holistic, addressing the entire educational ecosystem comprising also the actors, institutions, and regulations at all levels, as well as the ways in which one can intervene on these to facilitate the desired effect.

The structure of the research is divided in three main stages: identification and assessment of behavioral factors for recycling, identification and assessment of educational interventions for recycling and correlation of these components previously determined. The elements considered as constructs targeting behavioral changes as well as for educational interventions are acquired from a broad literature survey and processed by the means of interviews, focus groups and analysis tools and techniques. Their appraisal and correlation are derived by interviewing a large number of persons having relevant positions in the national and regional bodies responsible for education, in universities, schools and kindergartens as well as with people from companies delivering thematic training for adults. As processing and deployment tool, multiple QFD (Quality Function Deployment) analyses and a specialized software was used.

The first section provides a background for research, assembling a brief literature review on its essential components; the second one presents the steps of the research methodology along with the tools and instruments used, the next section describes the implementation of the methodology and the consequent discussions, while the last section offers the conclusions drawn and possible directions for future research.

## 2. Theoretical Background

### 2.1. Waste Management and Recycling

A fundamental idea of sustainable development is „Doing more with less” [12], which means decoupling the increase of environmental degradation from the economic growth and continuous expansion of consumption. Human behavior related to consumption and expectations for prosperity create worldwide a tremendous pressure on economy, resources and sustainable waste management.

The very recent report of World Bank [13] is mentioning for now 2.01 billion tons of Municipal Solid Waste (MSW) annually generated worldwide, from which more than a third is not managed in an environmentally safe manner. This total quantity is expected to grow with more than 70%, to 3.4 billion tons, by 2050, while in lower middle-income countries from regions such as Sub-Saharan Africa the increase is estimated to be more than three times and in South Asia up to twice the current level.

These results have to be considered in the context in which rich and developed countries recycle currently more than one third of their MSW, while in those with low-income over 90% of waste is openly dumped or burned [13]. It means that the need for a sustainable waste management will be higher in the areas that even now are the most vulnerable in this regard and the least prepared for solving the problems thereunder.

Concerning waste management itself or integrated in the circular economy as a comprehensive thinking model, programmatic documents of worldwide organizations as well as adopted by states or unions of states can be identified and cited, establishing directions and targets in this matter. One of the main waste management treatment measure is recycling [14], a solution to reduce the use of raw materials and amount of waste disposed on landfills. As an example, the known EU Directive 2008/98/EC on waste [15], that included targets for EU Member States to recycle 50% of their municipal waste by 2020, was recently amended by the Directive 2018/851 [16] that, among others, is renewing the mention target to: 55% by 2025, 60% by 2030 and 65% by 2035.

### 2.2. Theories Explaining People’s Behaviors, Particularly in Waste Management and Recycling

Since the beginning of 1970s, but moreover in the last decade, an increasing interest in research aiming to explain, predict or influence people’s behaviors toward environment was observed. At the beginning of this period, successful journals like Environment and Behavior (1968) and Journal of Environmental Psychology (1981) were instituted and new divisions dedicated to the environment of the well-known American Psychological Association and International Association of Applied Psychology were established [17].

Early research among which we recall [18] or [19], not as being the most significant but just to exemplify, mention the need for pro-environment modelling of human behavior and refers already to concepts such as *Knowledge*, *Awareness*, *Beliefs*, *Attitudes* as well as *Concern*, *Moral maturity*, *Motivation*, but without structuring these as constructs of a behavioral model.

In the last three decades, behavioral scientists increasingly turned their attention to the research of pro-environmental behavior, trying to explain it by means of experimental studies or surveys all over the world: Africa [20], South-America [21], Asia [22,23] or Europe [24,25], from various perspectives: targeting students [26] and households [27] or workplace behaviors [28].

One of the first theories used to explain the “ecological concerns” [29] is the Theory of Reasoned Action (TRA) developed by Fishbein & Ajzen [30]. According to TRA, the intention is the determinant of the behavior which in turn is influenced by the attitude towards that specific behavior and by the subjective norms [30]. Both attitude and subjective norms derive from an individual’s beliefs. The subjective norms are based on normative beliefs and motivation to comply—two elements influenced by the opinion of other important individuals or groups, while the attitude is more an expression of personal thinking and convictions [20], depending on the positive or negative views the people have regarding the consequences of the performed behavior (behavioral beliefs and outcomes evaluation) [31].

In 1991, Ajzen expanded TRA, with the concept of perceived behavioral control (PBC) resulting in the Theory of Planned Behavior (TPB) [32]. Independent of the attitude and subjective norms, PBC is based on the people’s perception about personal and environmental factors which facilitate or hinder their ability to perform a behavior—the so-called control beliefs [31]. Many empirical researches reported that PBC is the most significant predictor for the recycling behavior [33,34], but other constructs not included in TPB, such as information and knowledge are also important to form positive attitudes and intention towards recycling [35]. To be noted that the behaviors’ predictors can vary from region to region and TPB can be extended with additional constructs as long these are relevant [32].

Another theory applied to explore the pro-environmental and recycling behaviors is the Values Beliefs Norms (VBN) Theory which highlights the influencing relation of general values on specific behaviors [36,37], but is seen as less appropriate than TPB to predict the recycling behavior [38]. On the other hand, the Norm Activation Theory (NAT) emphasizes more the role of morality expressed through personal norms, which are feelings of moral obligation to behave in a certain way [39,40], whereas in TPB they are just one of the three main constructs to predict intention.

Some other papers, from which the quite recent [41] discuss motivations for pro-environmental behavior within a basic ABC analysis that places behavior B as a function of the antecedents A, that precede it and generating the consequences C, that follow it. Here above just some more common behavioral theories and models are cited as a framework for studying people’s behavior regarding waste management and recycling. Beyond these, the literature offers numerous other models extending the above mentioned ones or combining them with other theories or elements, or even proposing new, more or less original models that the authors tried to validate by empirical investigations.

In the authors’ opinion, the main added value of behavioral theories lie in identifying the primary factors that determine human behavior and in structuring them in a logical framework which leads, in a given configuration, to rational expectations regarding people’s behavior. The present study uses the first part of this contribution, namely the inventory of primary factors of human conducts from models applicable in pro-environmental area, which are then assessed for addressability by educational interventions.

### 2.3. Interventions for Changing Waste Management and Recycling Behaviors

The researches explaining people behaviors toward environment and waste recycling were usually focused on factors impacting these behaviors and on potential directions of intervention to facilitate those favorable to a sustainable development. Their final target was usually to identify which are the most effective and efficient (less expensive, stronger, with faster effects) directions of interventions or measures that can positively influence these behaviors.

An important number of research publications, books and reports focus on technical-managerial interventions concerning waste management and recycling. These [42,43,44] are targeting waste reduction and increasing the recycling rate by acting upon products on their life cycle as main waste sources, or upon waste management and technological processes such as waste collection, treatment and recycling.

The second category of publications [45,46,47] adopts as research subject the social factors influencing population priorities and responsible attitudes toward waste management and recycling. Besides moral motivation, direct economic rewards and penalties are taken into consideration [41].

Other studies support information dissemination and knowledge development on recycling as key factors for successful behavioral change [35,48], although information alone is not sufficient if the activity to be performed is not convenient, easy and inexpensive [49]. Informational interventions can’t always impact the behavior, even when the previous conditions are fulfilled and this is due to lack of motivation [50].

Since their beginning, the main directions for interventions identified by these researches were connected to environmental education which is considered to become a “must” for the modern society and the most consistent and effective path for its long-term sustainable development. The environmental education refers to all type of actions, campaigns or programs aiming to develop knowledge, attitudes and behaviors related to nature [51], as its aim is to make people act “in, with and for the environment” [52].

## 3. Research Methodology

### 3.1. Research Design

**Figure 1:**
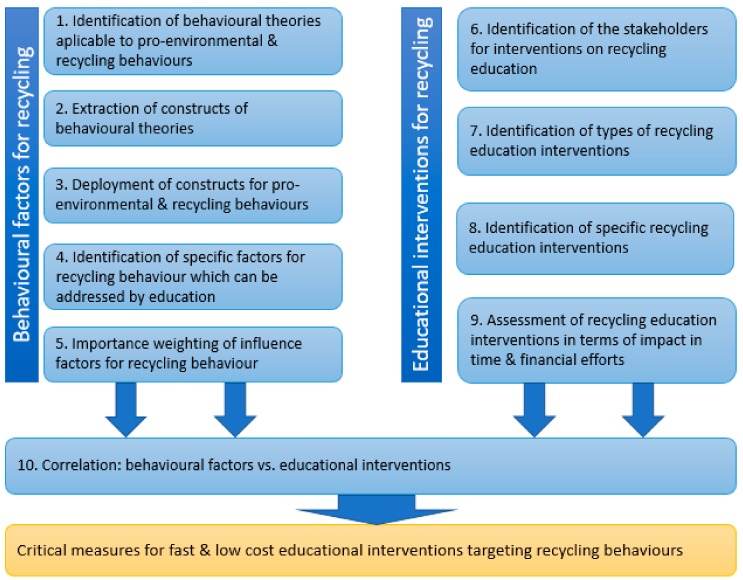
Research stages.

This research aimed to bring a contribution to the way concrete measures for fast, effective and low-cost educational interventions targeting development of recycling behaviors are conceived. The reasoning behind the elaboration of this research methodology was to define an adaptable approach easily applicable in similar topics such as environmental pollution or human health. The entire research demarche, as displayed in Figure 1, comprised three phases, the first two targeting its intermediary objectives in a parallel development using as roots two extensive literature surveys, followed by assessments within a professional framework of specialists in education, the outcomes being joined in a correlation to reach the final research goal.

The first intermediary research objective, to which correspond stages 1 to 5, has targeted the identification and weighting of the behavioral factors which influence recycling behaviors; the second intermediary objective, corresponding to stages 6–9, aimed to determine the educational interventions targeting recycling behaviors and to evaluate their effectiveness in terms of time and costs. Both of them are based on a detailed literature research and the assessment of professionals from the specific field. The last stage focused on the identification of critical measures for fast, effective and low-cost educational interventions targeting recycling behaviors by correlation of the behavioral factors with the educational interventions through the relationship matrix of the QFD method. A detailed step-by-step process is presented in the next section.

### 3.2. Research Stages and Instruments

Table 1 summarizes the research subphases and lists the tools and instruments used during the investigation. In the beginning, a broad literature survey was conducted in order to identify and organize the relevant bibliographic sources for designing a conceptual framework of the field and deriving the initial information needed in the later phases of the research. For determining the framework for potential educational interventions facilitating waste management, and recycling behavior in particular, and the subsequent influence factors on behavior, recycling attitudes and practices, the literature was explored searching for keywords like: behavioral model, behavioral change theory, waste management, recycling behavior, recycling intervention, environmental education, educational interventions, sustainable waste management, but was not limited to them. Next research stages focused on gathering, processing, assessing and discussing the input from educational professionals.

The stages presented below led to achieving the goal of the study—to formulate a set of critical measures for educational interventions targeting recycling behaviors answering simultaneously to three criteria: high impact, low cost, and fast results following a structured method which can be easily replicated. A detailed description of the work undertaken in each phase of the research is presented in the Section 4.

### 3.3. Briefing of Research Methods

The KJ technique, called also affinity diagram is a quality management tool developed by Kawakita Jiro, used for sorting and organizing numerous ideas into groups, based on their meaning [53]. It was used in this research for refining the set of influencing factors into the proper categories.

The questionnaire is one of the most popular research methods used for collecting data from respondents, with reduced efforts and providing a good base for analysis. On the other side, interviews can be more costly and time-consuming, but they reduce the chances that the respondents don’t understand the questions, so the authors used both methods in this investigation to ensure that accurate responses are collected. Due to resources restraints, this is a qualitative investigation therefore the respondents’ group was selected based on a non-probability sampling technique. Likewise, as a form of qualitative research, the focus group tool was used to gather professionals’ opinions in critical moments of the investigation. In this way, the analysis process of the influencing factors and educational interventions was improved and accelerated.

For processing the data gathered, techniques like clustering (for grouping items into categories), cost assessment (for evaluating direct and indirect costs) and paired comparison [54] were applied.

The Quality Function Deployment (QFD) is another tool from the quality management field, developed by Japanese Yoji Akao in 1960s and aimed at improving customers’ satisfaction by translating their demands into specific product characteristics in a structured and systematic way [55]. QFD’s “House of Quality” tool highlights the value adding features in meeting the needs of customer, which can be beneficial in making design decisions. After the main body containing the correlations between the requirements (“what”) and characteristics (“how”) is fulfilled by competent persons, the relative importance *w*′*_j_* of characteristics is calculated by multiplying the total of the sums of each column with the importance factor [56]:

(1) $${w^{\prime }}_{j}=\sum_{i=1}^{k}d\cdot r,\;j=1,2\ldots ,n.$$

where:

*w*′*_j_* = relative importance for design characteristic *j*th; *j* = 1, 2, …, *n*;

*d_i_* = degree of importance of the customer requirement *i*th, *i* = 1, 2, …, *m*;

*r_i,j_* = numerical relationship between customer requirement *i*th and design characteristic *j*th; *i* = 1, 2, …, *m*; *j* = 1, 2, …, *n*;

*m* = number of customer requirements;

*n* = number of design characteristics.

In the present research, QFD was applied to link the educational interventions with the factors of influence, for identifying the best correlations. The bottlenecks are defined as limitations that have to be addressed successfully to become critical items for achieving the target.

## 4. Research Description

### 4.1. Identification and Assessment of Behavioral Factors for Recycling

As previously mentioned, the present research explored some of the behavioral models used to explain recycling behavior with a main focus on TPB and its extended versions, in an attempt to find the factors which can lead to behavioral changes. Generally, the more powerful is the intention, the more probably is that the behavior of interest will be performed, but without the ability and the conditions to do it, the chances of it happening are decreasing considerably [31]. 

Due to complexity of the human nature, no theory can totally explain the predictors for a conduct, so this research tried to identify the main constructs from several theories and the underlying components applicable to recycling as found in the literature. The present study restricted its scope to the educational area, so from the factors previously identified, only the ones which can be addressed by education were selected, as considered by the questioned specialists. The outcome of these stages is displayed in Table 2.

The recycling behavior’s influencing factors from Table 2 were the subject of an evaluation process aiming to determine which of them can be influenced by education. This was performed within a focus group of four external participants, specialists selected from the educational field: 1 pre-university teacher, 2 university professors and 1 psychologist specialized in pre-school education. The influencing factors for recycling behavior were analyzed, rephrased and sorted using the KJ method, a quality management tool for structuring information, typically used within working groups.

**Figure 2:**
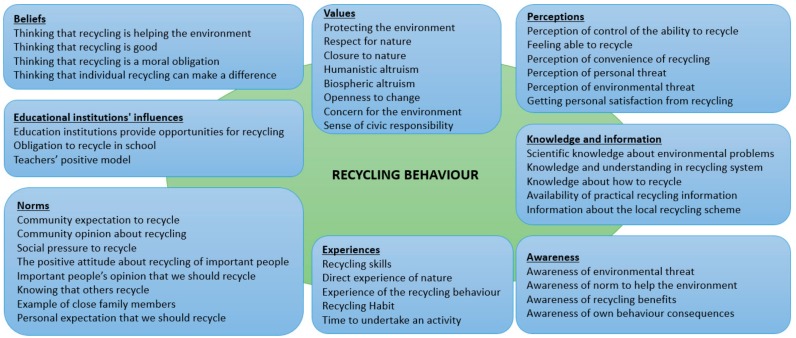
Influencing factors for recycling behavior which can be addressed by education.

The evaluation process within the focus group involved: presentation of all factors, discussion of their meaning, relevance for the topic and sensitivity to be influenced by education for each factor individually, rephrasing of the factors and their structuring into categories using the KJ method. During discussions, new factors were added and some of the existing ones were removed from the list being considered as less relevant. As result, a final list of 8 categories was established with a total of 43 influencing factors as displayed in the Figure 2 below.

The next step of the research was to assess the importance of these factors on developing recycling behavior, from an educational perspective, the goal being to obtain an objective and competent ranking, so it was decided to collect specialists’ point of view by conducting a questionnaire survey. The questionnaire design started form the identified factors and the respondents were asked to evaluate the importance of each factor for influencing recycling behavior on a Likert scale from 1 to 5, where 1—not important at all, 3—neither important or unimportant, 5—very important. The first draft included two sections—a short one with demographic questions and a major section which consisted of the main question (“Please select the statement which best expresses your opinion about the importance of each item in developing a recycling behavior”) and the list of all factors grouped by categories. This version was first pre-tested in a small group, being followed by a collaborative process where the authors discussed the observations and made changes which led to the final version of 2 identification questions (about occupation) and the main question asked about 36 specific items. The list was reduced from 43 to 36 factors, because some factors seemed redundant, or referring to same idea: humanistic altruism and biospheric altruism were considered together; perception of control of the ability to recycle, feeling able to recycle and recycling skills were merged; social pressure to recycle encompasses awareness of norms to help the environment and personal expectation that we should recycle while the following three items were merged into one: the positive attitude about recycling of important people, important people’s opinion that we should recycle and example of close family members.

The questionnaire was kept as simple as possible, using clear language to be easily understood by respondents. Using the Likert scale for the evaluation may raise some disadvantages, as the weighting depends on the understanding level of the participants and their perception, but these were minimized through rephrasing based on the pilot-phase outcome and through the careful selection of respondents.

The target group of respondents was defined as professionals from Romania working in the educational field with roles like educators, teachers, professors, trainers, inspectors, educational institutions’ managers or other administrative staff from the field. Due to characteristics of the present study (qualitative research, limited time and resources, difficulty to contact potential respondents), a non-probability sampling method was used—convenience sampling technique [65], so the respondents were chosen from known people and based on recommendations. There was conceived a list of 120 people, to fill in the questionnaire. A total number of 57 responses were received, representing a response rate of 47.5%.

The collected data was analyzed, targeting to determine a score for each factor—score to be used as input for the next research stage. Due to the objectives of the present paper, the results interpretation is limited to displaying only the factors identified as most relevant for developing a recycling behavior (Table 3).

### 4.2. Identification and Assessment of Educational Interventions Specific to Recycling

An important differentiation to be made here from the beginning, is between level of education as a demographic factor influencing environmental recycling behaviors (together with age, gender, etc.) and education as a major direction of intervention for developing positive behaviors toward environment and recycling.

The present study addresses improvements in the recycling behavior through different types of educational interventions targeting the implementation in educational institutions. These interventions are seen in a broad sense, addressing aspects related to education itself together with specific tools, education stakeholders, educational institutions or educational systems at different levels, with the final purpose of changing the targeted behavior. In this study, educational interventions are perceived beyond the educational content and tools; they are the means to create a framework favorable for behavioral changes.

First and foremost, it was needed to establish the subjects and the actors of the interventions, so a short literature screening was conducted to determine the stakeholders: pre-school children, school children, students, trainees, educators, teachers, professors, trainers, scholar inspectors, staff from public and private educational institutions, organizations, national administration.

The next part of the research was in fact a maturation process, which started from defining the types of educational interventions in a general way, which were afterwards used as basis for a literature study and a refinement with professionals evolving to the final list of educational interventions targeting recycling behavior. The initial classification of interventions was necessary in structuring the demarche and the way of working.

For defining the types of educational interventions, after a literature screening as basis for the interview preparation, the authors conducted three interviews with professionals from education asking for their views on what kind of measures can be implemented within the educational system, how to intervene regarding the learners and teachers and how can the institutions act. Their responses helped to clarify the major directions of intervention and to structure them in two major areas according to their subjects and their nature. After discussions, the authors decided on the categories and subcategories of interventions which are listed in Table 4.

The above classifications are not exhaustive or immutable, they are the result of the authors’ study of literature and discussions with professionals. They may contain overlaps, but this differentiation was considered a must in structuring and formulating the directions of action for the next phase of research.

The intervention types from the first classification presents the main aspects on which one can intervene on the subjects: learners, teachers or institutions. These refers to classes or lessons (curriculum), tools directly used by learners such as group projects, workshops, debates, etc. and on the other side the tools the teachers should apply for a successful pedagogical process (teaching methods and instruments). Evaluations address knowledges, skills and competences through exams, tests, inspections or specific performance indicators. Motivational measures are specific to each level: scholarships and prizes for children, salary increases or promotions in case of teachers or even intangible incentives like status and recognition. Designing interventions for educational institutions implies besides defining the target of the intervention, determining the methods to be used (normative, managerial, financial, technological and human resource-related). Deciding to make an institution more environment-friendly can be translated into changes in rules, in procedures, in the way resources are used, in planning or in criteria for internal evaluation of performance. These indirect measures will all lead to different degrees of behavioral changes of the administrative staff, academic staff and students. In the same way, educational measures from the financial sphere will determine changes, as these are one of the main motivational triggers. Offering scholarships can help children to continue education, salary increases can determine teachers to be more actively involved in the educational process, financial support can help teachers attend trainings and improve their competences, etc. One can’t discuss about educational interventions without having in mind the actors of the educational process, namely the academic staff. To improve their abilities, interventions on employment criteria, initial and continuous training, performance indicators or work recognition could be a solution. In this increasingly digitalized world, measures from the technological sphere should be also present, facilitating the educational process by using virtual and augmented reality in class, and web and mobile applications at home or for remote education.

The following stage advanced to a more detailed level, by identifying interventions specific for recycling education. In this regard, a structured study reflecting the categories from Table 4 was conducted to extract possible interventions from the literature, compiled in a list which served as discussion starting point for the second series of interviews with professionals. The three interviews conducted provided new insights on how one can intervene within education, clarified the ideas from the literature, extended the list of measures and classified them based on their type and addressability. Finally, discussions and analysis between the authors led to the following list comprising 45 items that clearly reflect the directions and measures targeting enhancement of recycling behaviors (displayed in Table 5). The Table 4 was important in structuring the demarche, representing the basis for classification but it was reconstructed during the process to be relevant for the specific recycling interventions.

In order to use the above list as input for the next research phase, a differentiation between items was needed and it was decided to assign each of them a difficulty index (displayed in Table 5) which incorporates these four criteria: cost, complexity of implementation (knowledge and skills required, dependencies, systems affected, etc.), number of people involved in the implementation and duration of the implementation.

The evaluation was realized by the means of a focus group consisting of 5 people (2 high school teachers, 2 professors and 1 trainer) who were first asked to rank the items on a scale from 1 to 10 (1—low costs/low complexity/reduced number of people involved/short time and 10—high costs/high complexity/high number of people involved/long time). Afterwards, all the participants discussed the significant differences and had to agree to the final scores. The average of the 4 scores for each item resulted in the difficulty index (displayed in Table 5), which was once again verified by the group. The focus group session was moderated by one of the authors which suggested some evaluation methods and intervened just for encouraging all the participants to express their opinions and ensure the purpose of the discussion was met, but without personal involvement.

The high scores in the categories of curricular interventions, teaching materials, regulation, legislation can be explained by the specificity of the investigation carried out in Romania where this type of changes involves a substantial effort both from a procedural aspect (content development, evaluation and approval) as well as costs (editing and publishing new textbooks). The methods and tools address a wide range of implementation options, from a base level (teacher/trainer) to a general level (national scale), hence the lower scores.

### 4.3. Correlation between the Behavioral Factors and the Recycling Educational Interventions

The next phase of the research consisted in the appraisal of the educational interventions from the perspective of the influencing factors and their impact, both determined on the basis of literature with an input from education professionals, having as final goal to support decision-making by providing an effective set of interventions for enhancing recycling behavior through education. The correlation process included the use of the QFD tool for analyzing data, a survey for collecting competent opinions and a focus group for processing the results.

For facilitating and automating the correlation process, the data gathered during previous research stages was inserted into the Quality Function Deployment tool—using the Qualica QFD software, with an adapted application to this case, entering the factors on the requirements’ side and the interventions on the characteristics’ side. The customization involved also removing the roof of the matrix considered unnecessary, as the interventions were already assessed for cross-correlation. For an improved analysis of the results, it was decided to break down the list of interventions into three parts (based on the categories identified) and to create three correlation matrices.

Once the House of Quality (the correlation matrix) was built in the tool, a print of it was prepared for distribution in order to collect scores of correlations from professionals. A group of 15 professionals from the educational field was selected to receive the correlation matrix, they were contacted asking them to evaluate to which extent the measures from the vertical side respond to the factors listed horizontally. Additional to the relations between the items, it was asked of them to consider the impact of the measures in terms of time until impact, impact size and broadness of impact (size of group addressed). Clear instructions for filling in the matrix were provided and the software’s relationship scale was: 0—no correlation, 1—possible correlation, 3—some correlation, 9—strong correlation. Moreover, respondents were invited to provide additional comments. With a certain insistence, 12 responses were collected.

The scores obtained from the survey have delivered the baseline of the focus group session formed by the group of authors, ensuring in this way complementary competencies for assessment of data. The resulting correlation matrices were compared by overlapping, the differences were discussed and analyzed which led in the end to a final score for each correlation. The correlation scores were introduced in the QFD tool and it was run through automatic calculation.

**Figure 3:**
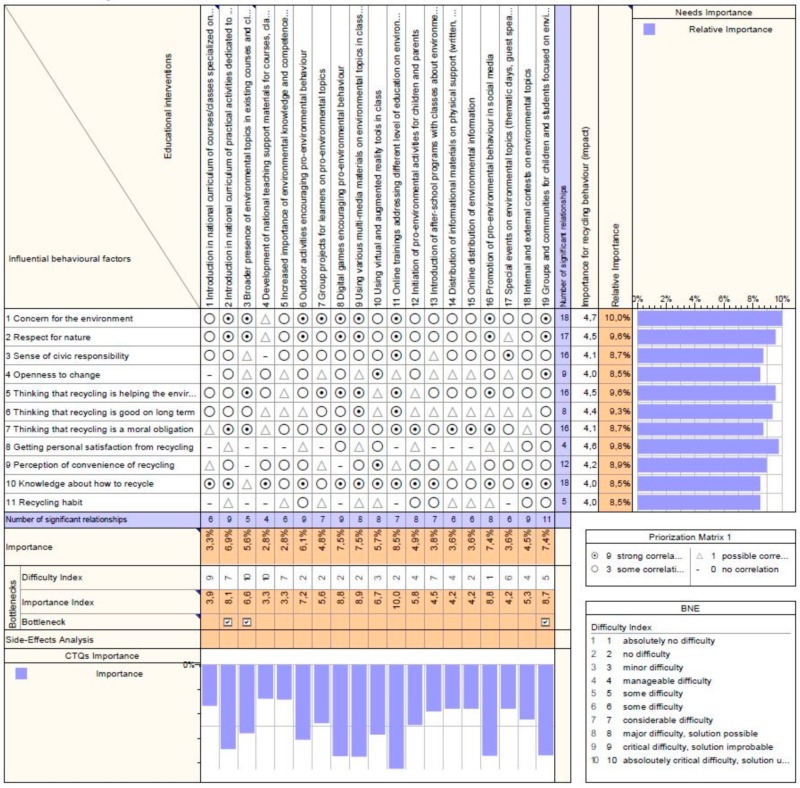
Correlation Matrix for interventions addressing learners—output from the Qualica QFD software.

**Figure 4:**
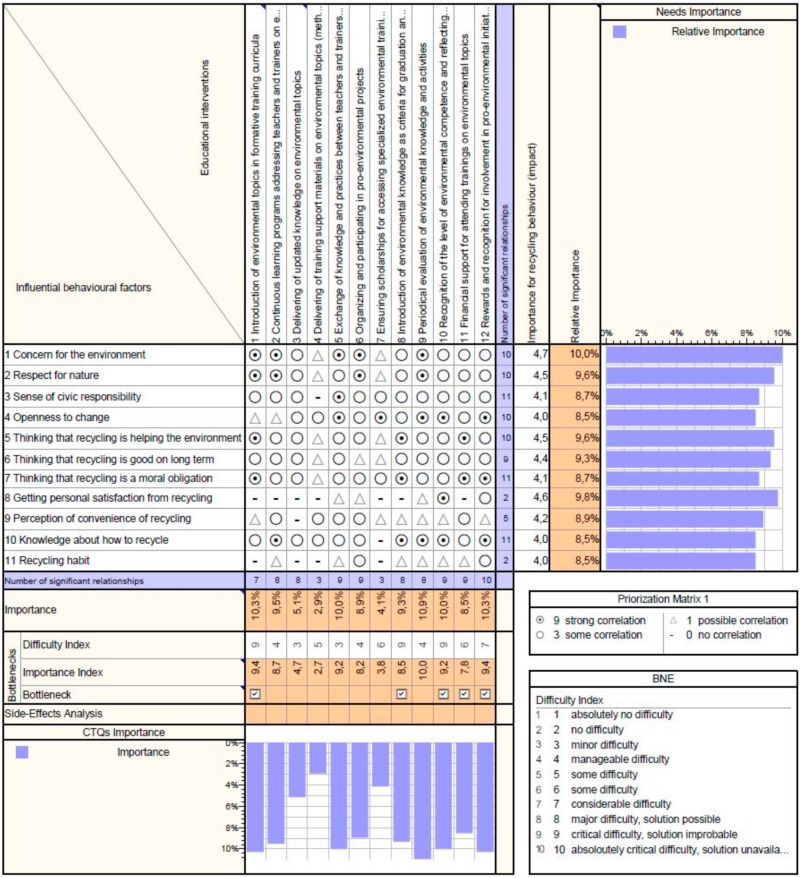
Correlation Matrix for interventions addressing teachers and trainers—output from the Qualica QFD software.

**Figure 5:**
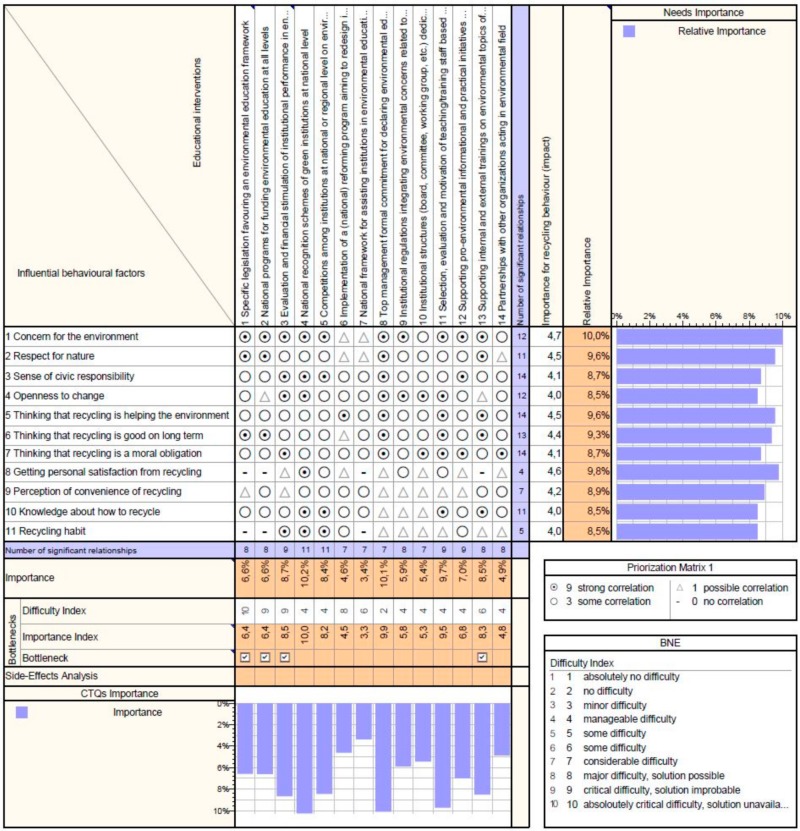
Correlation Matrix for interventions at national and institutional level—output from the Qualica QFD software.

The results are displayed in Figure 3, Figure 4 and Figure 5. These figures present an output from the Qualica QFD software used, where the scores of the influencing behavioral factors resulted from the questionnaire data are displayed on the right, in the column *Importance for recycling behavior (impact)* while in the next column *Relative importance* is the percentage representation of these scores. The difficulty index assigned to educational interventions during the focus group is shown on the bottom of the matrix. Based on correlations (filled in according to professionals’ opinions), the program calculates automatically the *Importance* and the *Importance index* of the interventions, which are then included in the analysis of bottlenecks along with the difficulty index.

As Figure 3 indicates, the top 5 interventions addressing learners are from the methods and tools category (all with an importance index over 8):

1. Online trainings addressing different level of education on environmental topics;
2. Using various multi-media materials on environmental topics in class (video, audio, etc.);
3. Digital games encouraging pro-environmental behavior;
4. Promotion of pro-environmental behavior in social media;
5. Groups and communities for children and students focused on environmental topics.

So, good results in changing recycling behavior seem to generate interventions from the digital area with fast and direct impact on the learners. Very low scores, three time less important as the top initiative were obtained for measures with a rather indirect effect on learners and consequently a long-term to impact (item 4—development of teaching support materials and item 5—increasing importance of environmental knowledge and competences in evaluation). As well, the informational interventions where the subjects are not directly involved in the educational activity are among the last in the ranking. Interestingly, organizing contests on environmental topics seems to bring fewer benefits than expected, despite the direct involvement of participants and the need for developing knowledge and competences in the field. One explanation could be the fact that such an initiative affects a reduced group of people, presumably the ones which already present interest in environmental topics.

Interventions like introduction in the national curriculum of practical activities dedicated to environmental topics and broader presence of environmental topics in existing courses and classes scored very well but are bottlenecks in the framework of present research due to their high difficulty index and long-term impact. Nevertheless, they should be considered for a consistent strategic approach in developing environmental education.

Generally, it can be affirmed that the interventions with a potentially broader impact and which imply direct involvement of educational subjects are in strong correlation with the development of recycling behavior.

The best ranked in the category of interventions addressing teachers and trainers are from the motivational sphere, both internal and external (Figure 4). These present good results but are mostly linked to high financial efforts or a high implementation difficulty, so if bottlenecks are eliminated the top interventions with importance index over 8 are:

1. Periodical evaluation of environmental knowledge and activities;
2. Exchange of knowledge and practices between teachers and trainers (workshops, open lectures, etc.);
3. Continuous learning programs addressing teachers and trainers on environmental topics;
4. Organizing and participating in pro-environmental projects.

Similar to the results in the previous category, it seems that measures dedicated to practical experiences are most effective in developing the knowledge and skills needed for promoting and teaching recycling. The informational measures are not linked to significant improvements in environmental knowledge and competences. Introduction of environmental topics in the mandatory formative programs (initial and continuous learning) brings significantly more benefits than just offering the choice which type of training to attend. In this regard, the two interventions (item 7—ensuring scholarships for specialized environmental trainings and item 11—financial support for attending trainings on environmental topics) differentiates themselves by the fact that the first has an optional character and covers only partially the financial efforts while the second one refers to choosing environmental topics from the list of obligatory trainings.

Although for teachers and trainers, as facilitators of education, it is a prerequisite to possess environmental knowledge and competences, this is not enough for adopting and for determining the development of students’ recycling behavior. There is a need also for motivation and financial support for transforming theoretical background into action, which can be ensured through institutional interventions.

The third correlation process led to the following list of top interventions (Figure 5):

1. National recognition schemes of green institutions;
2. Top management formal commitment to declaring environmental education as an institution’s priority (in strategical and operational plans);
3. Selection, evaluation and motivation of teaching/training staff based on pro-environmental knowledge and attitude;
4. Evaluation and financial stimulation of institutional performance in environmental education;
5. Supporting internal and external training on environmental topics of the institution’s staff.

These results are in line with previous ones highlighting the importance of motivating people involved in educational institutions to become promoters of recycling and pro-environmental behavior. The measures implying financial efforts are however bottlenecks and should be carefully considered in elaborating an improvement plan. Even the first ranked item could be argued if it can really bring the anticipated result without financial implications, as considered in this study.

It is not surprising that measures at institutional level aiming to create a culture favoring recycling starting from the top management commitment to selection of staff could have significant effects on recycling education: the facilitators of education should think and act pro-environmentally. Clearly, improvements on a large scale (national scale) could be achieved with measures targeting changes in the legislation and national funding program (bottlenecks), but these are extensive, costly and requires long time for implementation.

Overall, the correlation results highlight the most appropriate measures to improve recycling behaviors through education, measures with the highest impact on short term and on larger groups of people.

## 5. Discussion

The behavioral theories were used as conceptual background for understanding the recycling behavior and as basis for determining the factors which have the most influence in developing recycling behavior through education, resulting in a list of 43 factors from the following categories: values, beliefs, perceptions, awareness, knowledge and information, experiences, norms and educational institutions’ influences.

For the evaluation of the factors in terms of their impact on recycling behavior, input of professionals from the educational field was collected through a questionnaire, leading to a shortened list of just 11 factors, seen as relevant to be included in the next phase of the research, elements categorized as values, beliefs, perceptions, experiences, and knowledge and information. The best scores were in the groups of values and beliefs. These conclusions are just partially in accordance with the results of the study [66] which, besides beliefs about consequences, identifies environmental context and resources, knowledge and intentions as important determinants for recycling behavior. None of the factors from the category norms, awareness or institutions’ influences were included in the correlation, as based on evaluations they present lower influence on the recycling behavior. So, according to the study’s results, it seemed that focusing on shaping people’s set of values and beliefs could bring more benefits than addressing the intention to recycle. Considering that this research only tested to what extent the professionals think some interventions are effective, future research is needed to test whether this is indeed the case.

The second stage of the research was also grounded in a literature study meant to identify the types of educational interventions and specific interventions for recycling behavior. For processing the interventions in order to categorize and weight them, we once again gathered professionals’ opinion through interviews. Although in this way, the data was quantitatively reduced, it brought a gain on the qualitative side. The interventions identified were evaluated considering the required financial efforts, the complexity of the implementation, the number of people involved in implementation and the duration of it, all reflected in the difficulty index.

The correlations of the behavioral factors influencing education and the educational interventions for recycling behavior resulted in one set of critical measures for each of the three big categories: interventions addressing learners, interventions addressing teachers and trainers and interventions at the national and institutional level.

With regard to the critical interventions identified, the highest scores were obtained by measures from the digital area, which is easily explained by its main arguments: broad and fast impact, ease of use and popularity among young people. This is also not surprising, considering that digital learning is seen as achieving better positive effects on educational outcomes than the traditional way [67,68].

Next, the measures targeting development of knowledge and skills through practical experiences are valuable in the development of the recycling behavior, which is in line with the findings of the study by Oztekin et al. (2017) who see the past behavior as an important predictor of recycling [69] and connectedness to nature as significant for environmental education [70,71], but it is essential to integrate motivational measures to determine the educational facilitators to teach about it. Others have also shown that intrinsic and extrinsic motivations are very relevant in improving recycling behavior [72]. So, further research is encouraged to determine the motivational determinants and their predictive capacity in environmental education.

Of course, all these measures identified can’t be decoupled from the financial factor, which is a significant incentive, but solutions with reduced costs can be found: environmental knowledge as criteria for graduation and promotion, periodical evaluation of environmental knowledge or recognition for involvement in pro-environmental initiatives.

Certainly, a consistent process for improving recycling behavior through education should consider creating the institutional framework at national level favoring such changes: curriculum, legislation, national programs—reflected as bottlenecks in the results of the analysis. Bottlenecks are interventions with strong correlation, that also require increased effort to be implemented, situation which does not meet the immediate objectives of the present study. If impediments are addressed in order to reduce the difficulty index, therefore improving the effort vs. impact ratio, they could be effective measures to change recycling behavior.

For a multi-directional approach, the authors recommend including interventions from all the three categories, even subcategories as they present interdependencies which addressed together may lead to substantial modifications in recycling behavior and pro-environmental culture in the educational institutions. Moreover, it should be noted that a coherent approach with interventions on all the three dimensions should start from ensuring the institutional framework and support which enables teachers’ formation in a pro-environmental culture and consequently determines the application of the most effective teaching tools. Examples are numerous: support from institutions to organize projects, to attend trainings, to acquire new teaching methods or effective curricula changes which can’t be carried out without supporting teaching materials. In the absence of such a systematic endeavor, the results would be rather singular and wouldn’t yield a large scale impact.

The present research has some limitations, due to the reduced resources for collecting data from educational professionals, resulting only in qualitative information and presenting a high degree of subjectivity. Due to these restraints it is difficult to generalize the results. It is possible though to conduct similar research in a particular region or even within an institution for obtaining results with higher relevance, by providing the local character to the interventions which is seen as a need by [73]. Future research could focus on gathering feedback and lessons learned from practical experiences of implementing measures, based on which to improve the theoretical model.

Second, the list of interventions proposed is not exhaustive but reflects what was considered relevant by the professionals involved in this qualitative research. Definitely, it can be further adapted to specific cases or extended with additional interventions.

Next, the interventions identified were evaluated from a theoretical perspective based on personal experience and knowledge of the interviewed persons while no actual comparison data about their effectiveness on recycling behavior was used. A refined evaluation can propose an alternative set of measures, depending on the objectives established.

Nevertheless, the present study aimed to propose a structured approach to support decision regarding ways to improve recycling behavior by offering a prioritization method. The model presents the advantage of being simple and adaptable to the needs and available resources (financial, know-how, infrastructure, etc.). Further, based on the desired outcome the list of interventions can be reduced to only one category or extended with specific measures by repeating the application of the QFD technique to consecutive levels of detail. Being customizable, the model could be at any time extended to particular categories, specific factors, concrete interventions or the evaluations could consider only one critical dimension (for example the costs).

## 6. Conclusions

Considering the urgency of taking actions towards a more sustainable world everywhere on the planet and to start acting pro-environmentally, this study intended to answer to the challenges raised in this matter especially in countries where these interventions need to be not only with fast and with high impact, but also with low costs. Recycling behavior is one of the solutions to mitigate the waste management problems, so the target of this paper is to propose a way of determining the most efficient educational measures for improving recycling behavior.

The study of the literature showed that predictors of recycling behavior are complex and dependent of social, cultural and contextual factors and on the personal set of values and beliefs [24], so there is no unique solution for making people recycle. What the present study proposes is a structured approach, easily customizable to the needs and resources of the decisional factor targeting to improve recycling behavior through education. It can be applied both on a larger scale (national) and on a smaller scale (local, institutional), based on the objectives and resources for conducting the evaluations. As a methodology, it can be extended to any other thematic interventions addressing the behavioral change on particular issues such as environment, health, or any other considered as being important for the common interests of society.

First, understanding the factors which can positively influence the recycling behavior may be one of the starting points to develop solutions for improving this behavior. In this regard, TPB and extended theories based on TPB provide a good theoretical background for identifying the constructs framing the recycling behavior. Although many studies see convenience as one very important predictor or the lack of it as a barrier for recycling, this research limits the interventions to the ones which can be implemented in educational institutions. Next, we obtained professional input from specialists from the educational field through a questionnaire based survey meant to evaluate and rank the factors identified resulting in a shortened list of the most influential factors for the recycling behavior, which is one of the contributions of this paper. These, together with the specific educational interventions, once again based on literature and on the input of professionals, build the theoretical framework which is the main contribution. This study is focused on measures within educational institutions because education was considered the most powerful instrument to change people’s behavior. The key stage in establishing a set of critical interventions in education to achieve the optimum mix of high impact, short term results and low costs, was the correlation process which involved, using an advanced tool of competitive development (QFD), the input from education professionals and the contribution of authors for the refinement of the results.

Despite its applicative character, this study has some limitations, regarding the lack of empirical evidence and its subjectivity, being primarily based on literature review and professionals’ experiences and know-how, without investigations of the actual effects of the factors and interventions identified. Testing the effectiveness of the educational interventions in practice, to a reasonable scale can be the object of future research.

The present research does not offer a generally valid solution, but it proposes a way of developing the solution, a customizable approach, where the specific characteristics of the target can be included for obtaining effective behavioral change interventions. The educational interventions taken into account were structured and limited to those using institutional/organizational channels addressing clearly identified groups of people. Another interesting future extension of this research could be the identification of efficient educational interventions addressing a large, unstructured population using media and online facilities.

As mentioned before, the present demarche could be extended in the future, by tailoring the components to contextual requirements, for a wide range of researches aiming to change people’s behavior in other areas of public interest (including environment and health issues) through practical interventions, formulating policies or rethinking in-place educational programs.

## Figures and Tables

**Table 1:** Summary of research subphases and tools & instruments.

	Research Subphase	Subphase Summary	Types of Tools and Instruments
Behavioral Factors of Recycling	0a. Searching, structuring and filtering the relevant literature	- Search for relevant publications in the main databases (ScienceDirect, Web of Knowledge, Google Scholar) using keywords such as: behavioral model, behavioral change theory, waste management, recycling behavior, sustainable waste management, pro-environmental behavior, pro-environmental attitudes, pro-environmental values, learning models. Search extension for other papers within the reference lists of previously found, reaching a list of 19 books and more than 100 scientific papers and reports as basis for the next filtration phase. - Structuring of references by topics (e.g., behavioral models, changing behaviors, pro-environmental attitudes, values knowledge and behaviors, factors influencing behaviors, etc.) and selection of the most relevant for each topic.	- Keyword search - Bibliographic mining - Organizing techniques
	1. Identification of behavioral theories applicable to pro-environmental and recycling behaviors	- A critical study of the resulted literature led to a list of the behavioral theories applicable to pro-environmental & recycling behaviors	- Literature study
	2. Extraction of constructs of behavioral theories	- Identifications of the constructs from the identified behavioral theories. - Listing of constructs in a centralized table.	- Literature review - Data extraction
	3. Deployment of constructs for pro-environmental and recycling behaviors	- Identification of recycling behavioral factors from the associations made within the selected literature between constructs of behavioral theories and pro-environmental and recycling behaviors. - Listing these factors to be later evaluated if they can be influenced by education.	- Data extraction - Data analysis
	4. Identification of specific elements of pro-environmental and recycling behaviors addressable by education	- Discussion, assessment and structuring in categories of the identified factors, by means of a focus group. - List of behavioral factors sensitive to be influenced by education grouped by categories.	- Data analysis - KJ/Affinity Method - Focus group
	5. Importance weighting of influence factors for recycling behavior	- Design of the questionnaire. - Pre-test and refinement of the questionnaire. - Definition of the respondents’ sample. - Distribution of the questionnaire. - Results’ collection. - Results’ analysis. - Ranking of the influence factors.	- Questionnaire - Sampling methods - Questionnaire analysis - Ranking methods
Educational Interventions	0b. Searching, structuring and filtering the relevant literature	- Search for relevant publications in the main databases using keywords such as: environmental education, recycling behavior, recycling intervention, behavioral intervention, information intervention, education intervention, influence of education, environmental curriculum, recycling program, education for sustainable development. Search extension for other papers within the reference lists of previously found, reaching an initial list of 136 books, conference and journal articles, handbooks and reports. - Structuring of the references by topics (e.g., environmental education, recycling interventions, education for sustainable waste management, informational and educational interventions etc.) and selection of the most relevant for each topic.	- Keyword search - Bibliographic mining - Organizing techniques
	6. Identification of the stakeholders for interventions on recycling education	- Identification and listing of relevant stakeholders for educational interventions (subjects and actors).	- Reference screening - Data extraction
	7. Identification of types of educational interventions	- Identification of educational interventions types. - Three interviews with professionals from the education field. - Listing in a centralized table with general types of interventions.	- Reference screening - Data extraction - Interviews
	8. Identification of specific pro-environmental and recycling educational interventions	- Identification of specific interventions for pro-environmental and recycling education from literature. - Analysis and classification of the interventions. - Three interviews with professionals to process and discuss the interventions. - List of 45 educational interventions.	- Literature review - Classification technique: clustering - Interviews
	9. Assessment of recycling educational interventions	- Definition of an evaluation criteria for interventions (difficulty index). - Assessment of interventions within a focus group in terms of cost, complexity of implementation, number of people involved in implementation and duration of implementation. - Assignment of the difficulty index to interventions.	- Focus group - Analysis - Evaluation methods: cost assessment, paired comparison - Ranking methods
	10. Correlation of behavioral factors vs. recycling educational interventions	- Data entering in the QFD tool. - Preparation of the correlation matrix. - Collection of opinions from professionals regarding correlations. - Processing of data gathered within a focus group. - Formulation of the set of critical measures for educational interventions targeting recycling behaviors answering simultaneously to three criteria: high impact, low cost and fast results.	- QFD - Survey - Focus group

**Table 2:** Recycling behavioral constructs and factors which can be influenced by education.

Reference	Behavioral Model	Constructs Applicable to Recycling Behaviors	Recycling Behavior’s Factors to be Evaluated if They Can Be Influenced by Education
[57]	Own developed framework	Situational & psychological variables	change acceptance; closeness to nature; individual knowledge; experience of the behavior in question; awareness (all types); perception of convenience; intrinsic motivation; perception of personal threat; models (others’ behavior); self-efficacy.
[58]	TPB + socio-demographic, economic and situational factors—integrated perspective	TPB constructs Social norms Socio-demographic, economic and situational factors	perceived adequacy of recycling information; perceived recycling skill; attitude toward recycling; external subjective norm; awareness of recycling benefit; influence by norms of the involved societies; understanding of the recycling system; recycling skills; positive attitude; awareness of recycling; communities’ norms.
[59]	Adapted model based on TPB, extended with sustainability knowledge and values	Three global constructs (TPB) Sustainability values Sustainability knowledge Extension of attitude construct with three types: individual, local and global	knowledge: problem-based knowledge and solution-based knowledge; values; knowledge about economic, ecological, and social consequences of a given behavior and immediate and long-term consequences of a given behavior.
[60]	Causal relationships between environmental values and recycling behavior	Environmental values Behaviors intentions (mediating construct)	environmental values; moral norms; culture.
[61]	Extended TPB	TPB constructs Additional predicting variables: past recycling behavior, situational factors, and consequences of recycling, Demographic factors	attitude; awareness of consequences; past recycling behavior.
[62]	Conceptual framework based on Fishbein and Ajzen (1975)	Attitude Intention Behavior Environmental values Situational variables Psychological factors	environmental values; situational factors (contextual, socio-demographic, knowledge based, and experience based); psychological factors: altruistic motives, intrinsic and extrinsic motivations, perception of environmental threat, outcome beliefs, subjective norms; knowledge of how and where to recycle; perceiving that recycling is easy and convenient; the influence of others.
[24]	Study to explain the attitude—behavior gap	Relationships Personality Practicality Responsibility Culture	information about recycling; practical information; pessimistic or optimistic personality traits (believing that individual recycling can make a difference); sense of civic responsibility; socio-cultural norms.
[63]	Review of social norms	Social norms Habit Identity	visibility of recycling; social pressure exerted by the knowledge that others recycle; personal norms (expectation that we should recycle); pro-environmental values; social norm; habit; clear information.
[64]	Focus on the relationship attitude—behavior	Attitudes and behaviors	person’s informal, personal and intimate relationships and experiences; past and present formal education; formal formative influences.
[20]	TPB giving to each construct particular items for evaluation	TPB constructs	people should feel in control of their ability to recycle; habits; co-responsibility; moral values (injunctive norms); information about available recycling schemes.

**Table 3:** Most relevant influencing factors for recycling behavior based on questionnaire results.

Factor	Score
Concern for the environment	4.7
Respect for nature	4.5
Sense of civic responsibility	4.1
Openness to change	4
Thinking that recycling is helping the environment	4.5
Thinking that recycling is good on long term	4.4
Thinking that recycling is a moral obligation	4.1
Getting personal satisfaction from recycling	4.6
Perception of convenience of recycling	4.2
Knowledge about how to recycle	4
Recycling habit	4

**Table 4:** Classification of educational interventions.

Types of Educational Interventions				
***By the subject of the intervention***				
**Interventions at learners’ level: pre-school & school children, students, trainees**			**Interventions at teachers’/professors’/trainers’ level**	**Interventions at institutions level**
Curriculum			Curriculum	Regulations
Training structure and duration			Training structure and duration	Control and monitoring
Teaching methods and instruments			Teaching methods and instruments	Evaluation
Modern technologies			Modern technologies	
Evaluation			Evaluation	
Motivation			Motivation	
***By the nature of the intervention***				
**Normative**	**Managerial**	**Financial**	**Technological**	**Human resource**
Regulation	Management	Salary	E-learning (also mobile applications)	Selection
Legislation	Organizational strategy	Scholarship	Virtual reality & augmented reality	Initial training
Procedures	Resources	Institutional funding	Remote education	Continuous training
	Planning	Projects	Artificial intelligence	Performance assessment
	Monitoring and evaluation			Rewards and incentives
	Continuous improvement			Promotion

**Table 5:** Types of educational interventions and measures for pro-environmental and recycling behavior.

Types of Educational Interventions and Measures for Pro-Environmental and Recycling Behavior		Difficulty Index
**I. Interventions addressing learners**		
** A. Curriculum and contents**		
1	Introduction in national curriculum of courses/classes specialized on environmental topics	9
2	Introduction in national curriculum of practical activities dedicated to environmental topics	7
3	Broader presence of environmental topics in existing courses and classes	10
4	Development of national teaching support materials for courses, classes and lessons on environmental topics	10
5	Increased importance of environmental knowledge and competences in students and children evaluation	7
** B. Methods and tools**		
6	Outdoor activities encouraging pro-environmental behavior	2
7	Group projects for learners on environmental topics	2
8	Digital games encouraging pro-environmental behavior	2
9	Using various multi-media materials on environmental topics in class (video, audio, etc.)	2
10	Using virtual and augmented reality tools in class	3
11	Online trainings addressing different level of education on environmental topics	2
12	Initiation of pro-environmental activities for children and parents	4
13	Introduction of after-school programs with classes about environmental protection and waste management topics	7
14	Distribution of informational materials on physical support (written, audio, video)	4
15	Online distribution of environmental information	2
16	Promotion of pro-environmental behavior in social media	1
17	Special events on environmental topics (thematic days, guest speakers, conferences, etc.)	6
18	Internal and external contests on environmental topics	4
19	Groups and communities for children and students focused on environmental topics	5
**II. Interventions addressing teachers and trainers**		
** A. Acquirement of specific knowledge and skills**		
1	Introduction of environmental topics in formative training curricula	9
2	Continuous learning programs addressing teachers and trainers on environmental topics	4
3	Delivering of updated knowledge on environmental topics	3
4	Delivering of training support materials on environmental topics (methods, teaching tools, theoretical and practical training ideas)	5
5	Exchange of knowledge and practices between teachers and trainers (workshops, open lectures, etc.)	3
6	Organizing and participating in pro-environmental projects	4
7	Ensuring scholarships for accessing specialized environmental trainings	6
** B. Motivation**		
8	Introduction of environmental knowledge as criteria for graduation and promotion	9
9	Periodical evaluation of environmental knowledge and activities	4
10	Recognition of the level of environmental competence and reflecting it in the salary	9
11	Financial support for attending trainings on environmental topics	6
12	Rewards and recognition for involvement in pro-environmental initiatives	7
**III. National/Institutional level interventions**		
** A. National level**		
1	Specific legislation favoring an environmental education framework	10
2	National programs for funding environmental education at all levels	9
3	Evaluation and financial stimulation of institutional performance in environmental education	9
4	National recognition schemes of green institutions	4
5	Competitions among institutions at national or regional level on environmental topics	4
6	Implementation of a (national) reforming program aiming to redesign institutions recycling schemes	8
7	National framework for assisting institutions in environmental education (physical support, virtual assistance)	6
** B. Institutional level**		
8	Top management formal commitment to declaring environmental education as an institution’s priority (in strategical and operational plans)	2
9	Institutional regulations integrating environmental concerns related to staff, processes and outputs	4
10	Institutional structures (board, committee, working group, etc.) dedicated to environmental issues	5
11	Selection, evaluation and motivation of teaching/training staff based on pro-environmental knowledge and attitude	4
12	Supporting pro-environmental informational and practical initiatives within the institution	4
13	Supporting internal and external training on environmental topics of the institution’s staff	6
14	Partnerships with other organizations acting in environmental field	4
